# Downregulation of Transcription Factor T-Bet as a Protective Strategy in Monosodium Urate-Induced Gouty Inflammation

**DOI:** 10.3389/fimmu.2019.01199

**Published:** 2019-05-29

**Authors:** Qi-Bin Yang, Yong-Long He, Quan-Bo Zhang, Qing-Sheng Mi, Jing-Guo Zhou

**Affiliations:** ^1^Department of Rheumatology and Immunology, Affiliated Hospital of North Sichuan Medical College, Nanchong, China; ^2^Clinical Medical School, Chengdu University of Traditional Chinese Medicine, Chengdu, China; ^3^Department of Gerontology, Affiliated Hospital of North Sichuan Medical College, Nanchong, China; ^4^Henry Ford Immunology Program, Henry Ford Health System, Detroit, MI, United States; ^5^Department of Rheumatology and Immunology, The First Affiliated Hospital of Chengdu Medical College, Chengdu, China

**Keywords:** T-bet, monosodium urate, gout, inflammation, cytokines

## Abstract

Gout is sterile joint inflammation triggered by the damaging effects of monosodium urate (MSU) crystals accumulation. Previous studies suggest transcription factor T-bet plays an important role in inflammatory arthritis. Notably, mice lacking T-bet markedly reduced joint inflammation of rheumatoid arthritis models, however, the involvement of T-bet in gouty inflammation has yet to be clarified. Here, we took advantage of T-bet knockout (KO) mice to investigate the role of T-bet in the pathogenesis of MSU-induced gout inflammation. T-bet KO and wild type (WT) mice were used for models of acute inflammation induced with MSU crystals, including footpad, air pouch and peritonitis models. Inflammatory cytokines and phagocytosis were detected in bone-marrow-derived macrophages (BMDMs) from T-bet KO and WT mice treated with MSU crystals *in vitro*. In addition, T-bet expression in peripheral blood mononuclear cells (PBMCs) from gout patients was measured, as well as plasma inflammatory cytokines. We found that the levels of interleukin (IL)-17, IL-23, and interferon-γ were reduced, but tumor necrosis factor-α was not, in BMDMs from T-bet KO compared with WT mice after MSU challenge *in vitro*, as well as MSU phagocytosis. In comparison with WT mice *in vivo*, the swelling index of T-bet KO mice was significantly decreased in the footpad model. T-bet deficiency also dramatically relieved MSU-induced inflammatory cell infiltration in peritonitis and air pouch models *in vivo*, and as well as the IL-1β levels of air pouch lavage fluid (APLF). In addition, plasma IL-17 and IL-23 levels were elevated in acute gout, whereas protein levels of T-bet were downregulated in PBMCs from acute gout patients and intercritical gout treated with MSU crystals *in vitro* as well. Transcription factor T-bet deficiency protects against MSU-induced gouty inflammation, suggesting that downregulation of T-bet could be a protective strategy and contribute to spontaneous remission of inflammation in acute gout.

## Introduction

Gout is sterile joint inflammation triggered by the damaging effects of monosodium urate (MSU) crystals accumulation in the synovial space, which often manifests as an episode of acute and intense painful arthritis and involves a multicellular inflammatory process, including infiltration of macrophages, neutrophils, and lymphocytes. MSU crystals trigger NOD-like receptor pyrin domain-containing protein (NLRP)3 inflammasome activation in macrophages and mediate release of interleukin (IL)-1β, which is a crucial cytokine in acute gout, and neutrophils recruitment (1, 2). Neutrophils infiltrating the inflamed joints encounter and recognize MSU crystals, which results in their activation and subsequent amplification of inflammation. Currently, it suggests that innate immunity is involved in the pathogenesis of acute gouty inflammation, however, adaptive immunity is rare.

Transcription factor T-bet (encoded by *Tbx21*) is an immune-cell-specific member of the T-box family of transcription factors, which is best known for its role in T helper (Th)1-cell differentiation, but now recognized as having an important role in many cells of the adaptive and innate immune systems (3). A number of studies have reported that T-bet plays a major role in mediating autoimmune diseases (4). Mice lacking T-bet are protected from the development of many different Th1-driven autoimmune diseases (4), including systemic lupus erythematosus, multiple sclerosis, inflammatory bowel disease, and even collagen antibody-induced arthritis (CAIA). T-bet-deficient mice with CAIA produce less of inflammatory mediators interleukin (IL)-1α, CC-chemokine ligand (CCL)3, IL-17, interferon (INF)-γ (5, 6), which are involved in recruitment and activation of inflammatory cells such as macrophages, monocytes, and neutrophils. T-bet also directs T-cell homing to proinflammatory sites by regulation of chemokine receptor CXCR3 expression (7), which is also able to regulate leukocyte trafficking to sites of inflammation. In addition, a recent study from Lau et al. (8) reported that T-bet expression was increased in patients with ankylosing spondylitis (AS), however, the peripheral joint inflammation of AS model was significantly reduced in T-bet-deficient mice compared with wild type (WT) mice. The conflicting findings were consistent with most of the data from other typical autoimmune diseases (4). It remains unclear what mechanisms control the T-bet expression giving rise to the opposite outcome. Nonetheless, these findings suggest that T-bet plays an important role in inflammatory arthritis. Considering gout serves as a typical inflammatory arthritis and has a unique self-relieving characteristic, the involvement of T-bet in gouty inflammation has yet to be clarified.

In the present study, we took advantage of T-bet knockout (KO) mice to evaluate the role of T-bet in MSU-induced gout *in vivo* and *in vitro*. Diverse MSU-induced murine gout models, including footpad, air pouch, and peritoneal cavity models, were created. The inflammatory cytokines and phagocytosis of bone-marrow-derived macrophages (BMDMs) from T-bet KO mice treated with MSU crystals and cytokine IL-1β levels in air pouch lavage fluid (APLF) were measured. In addition, to confirm the role of T-bet in gout patients, T-bet expression in peripheral blood mononuclear cells (PBMCs) from patients with gout was measured as well as intercritical gout treated with MSU crystal to induce acute gout inflammation *in vitro*. Besides, plasma inflammatory cytokines in gout patients were also investigated.

## Materials and Methods

### Patients

Gout patients were included in the current study from the Department of Rheumatology of The Affiliated Hospital of North Sichuan Medical College. The acute gout (AG) patients had to meet the classification criteria of the American College of Rheumatology (1977) (9). Intercritical gout (IG) was defined as complete remission of AG and normal C-reactive protein or erythrocyte sedimentation rate. In addition, patients had no history of cancer, hematopathy, nephropathy, infection, or other autoimmune diseases. Age-matched men served as the healthy controls (HCs) who underwent regular physical examination at the Affiliated Hospital of North Sichuan Medical College during the same period. The characteristics of gout patients were summarized in Table 1. Blood samples were collected from them for analysis. The study was conducted in accordance with the principles of the Declaration of Helsinki and approved by the institutional research ethics committee of Affiliated Hospital of North Sichuan Medical College, and written informed consent was obtained from each subject.

**Table 1:** Characteristics of gout patients and healthy controls.

**Items**	**Acute gout**	**Intercritical gout**	**Healthy control**
Cases no.	45	42	42
Gender	Male	Male	Male
Age (years)	44.5 ± 9.1	42.2 ± 13.6	45.7 ± 11.3
Disease duration (years)	5.8 ± 3.1	7.3 ± 4.6	–

### Animals

T-bet KO mice on a C57BL/6 background and C57BL/6 as WT mice were purchased from Jackson Laboratory. The mice were housed at 24 ± 2°C under a 12-h light/dark cycle in a pathogen-free facility. Male mice aged 8–10 weeks were used to perform the experiments. Handling of mice and experimental procedures were approved by Institutional Animal Care and Use Committee of Henry Ford Health System.

### Preparation of MSU Crystals

MSU crystals were prepared as described previously (10). Briefly, 1.0 g of uric acid (Sigma–Aldrich) was dissolved in 200 mL boiling distilled water containing 6.0 mL 1 M NaOH. After adjusting the pH of the solution to 7.2 with HCl, crystals that formed were sterilized by heating at 180°C for 2 h. The solution was gradually cooled by stirring at room temperature and stored overnight at 4°C. The precipitate was filtered from the solution, dried under low heat, and suspended in phosphate-buffered saline (PBS) at a concentration of 50 mg/mL. All reagents were prepared under pyrogen-free conditions.

### MSU Stimulation and PBMCs Harvest

PBMCs (6 × 10^6^ cells) were isolated from gout patients or HCs using Ficoll–Hypaque density gradient centrifugation (Tianjin Haoyang Biotechnology, China). PBMCs (3 × 10^6^ cells) were divided equally and stored at −80°C for subsequent measurement of genes or proteins. In addition, peripheral venous blood from 4 patients with IG was treated with MSU crystals suspension (100 μg/mL final concentration), and then incubated in a humidified incubator at 37°C in 5% CO_2_ for 0, 3, 6, 9, or 12 h. PBMCs (3 × 10^6^ cells) were harvested according to the above method and stored at −80°C for subsequent measurement of proteins. The cells (5 × 10^5^ cells) from each group were analyzed by flow cytometry for assessment of viability with the Annexin V-FITC/PI apoptosis detection kit (Beyotime Biotechnology).

### BMDM Culture and MSU Phagocytosis in Mice

Bone-marrow cells obtained from femoral bones of T-bet KO or WT mice were cultured in RPMI with 10% fetal bovine serum and 30 ng/mL macrophage colony-stimulating factor for 7 days to induce BMDMs. Phenotypic validation of BMDMs was performed by flow cytometry with F4/80-PE (phycoerythrin) and CD11b-FITC (fluorescein isothiocyanate) staining. The purity of BMDMs was accurately assessed by a double positive for F4/80 and CD11b. For MSU phagocytosis of macrophages, BMDMs were harvested at 2 or 4 h after MSU crystal challenge. Phagocytosis of MSU crystals was determined by analyzing the side scatter (SSC) change in flow cytometry.

### MSU-Induced Gout Model

Mice were anesthetized (150:10 mg/kg ketamine: xylazine intraperitoneally) and injected with MSU crystals into the right footpad (1 mg in 40 μL PBS). The same volume of sterile PBS was injected into the other footpad at the same time as a control. Following MSU crystal injection, Paw swelling as inflammation parameters was measured with an electronic caliper at different time points (3, 6, 24, 48, and 72 h) (11–13).

Injection of 5 mL sterile air into the subcutaneous tissue on the back of mice to form an air pouch was followed by injection of an additional 3 mL air on days 3 and 5. On day 7, MSU crystal suspension (3 mg in 1 mL PBS) was injected into the air pouch, as described previously (14). For the MSU-induced peritonitis model, MSU crystals (3 mg in 0.5 mL PBS) were injected into the peritoneal cavity, as described previously (15). The air pouch and peritoneal cavity exudate cells with 2 mL PBS were harvested at 3, 6, 12, or 24 h for flow cytometry.

### Air Pouch and Peritoneal Cavity Cells Analysis

The harvested cells (1 × 10^6^) from the air pouch or peritoneal cavity were washed twice with staining buffer and incubated with Fc block (clone 2.4G2) for 15 min. The following conjugated monoclonal antibodies were used for flow cytometry analysis: neutrophil-Ly6G-PE, macrophage-CD11b-Perp-Cy5.5, macrophage-F4/80-FITC, T cell-TCRβ-ef450, and B cell-B220-APC-Cy7.

### Cytokine Assessment

For the inflammatory cytokines assessment, MSU crystal suspension was added to the incubated BMDMs (MSU 100 μg/mL final concentration) for 0, 4, or 8 h. The cytokines were measured by flow cytometry with the following conjugated monoclonal antibodies: TNF-α-PE-Cy7, IL-23-ef450, IL-17-APC, and IFN-γ-CD11b-Perp-Cy5.5. Dead cells were first gated out by propidium iodide (PI) staining. All data were acquired by CellQuest software (BD Biosciences) and analyzed by FlowJo software (Tree Star Inc.).

### RNA Extraction and Quantitative Real-Time PCR

PBMCs were isolated as previously mentioned and stored at −80°C until analysis. Total RNA (200 ng) was extracted from human PBMCs (3 × 10^6^ cells) using TRIzol reagent (Invitrogen) and reverse-transcribed into cDNA using reverse transcription reagents (TaKaRa). T-bet, and β-actin as an internal reference, were measured by qPCR. qPCR was performed with the SYBR Green I Two-Step qRT-PCR kit with ROX (Invitrogen). The gene primer sequences were synthesized by Sangon Biotech. The primers were as follows: T-bet sense, 5′-GATGTTTGTGGACGTGGTCTTG-3′ and antisense, 5′- CTTTCCACACTGCACCCACTT-3′; β-actin sense, 5′-GAGCTACGAGCTGCCTGACG-3′ and antisense, 5′-GTAGTTTCGTGGATGCCACAG-3′. Each sample was detected in duplicate and the average values taken. Gene expression was analyzed using the 2^−ΔΔCT^ method.

### Western Blotting

Proteins of PBMCs (3 × 10^6^ cells) were extracted by RIPA lysis buffer (Thermo Scientific) containing protease or phosphatase inhibitors. Proteins were quantified with BCA protein assay kit (Beyotime Biotechnology). The proteins (50 μg) were separated by 10% sodium dodecyl sulfate polyacrylamide gel electrophoresis (SDS-PAGE) and transferred to a polyvinylidene fluoride (PVDF) membrane (Bio-Rad). The PVDF membrane was blocked in 5% non-fat milk for 1 h at room temperature and incubated with primary antibodies [anti-T-bet antibody (#RLN2921, Ruiying Biological) and anti-β-actin antibody (#5125, Cell Signaling Technology)] at 4°C overnight. The secondary antibody was conjugated to horseradish peroxidase and incubated for 1 h at room temperature. Immunoreactive proteins were visualized using an enhanced chemiluminescence system (Engreen Biosystem). The PVDF membrane was scanned (Bio-Rad) and saved as gray scale image. Fold changes were assessed using Image J software.

### Enzyme-Linked Immunosorbent Assays

After centrifugation, plasma samples of gout patients or HCs were collected and stored at −80°C for cytokine assessment. IL-17 (Cat#SEA063Hu) and IL-23 (Cat#SEA384Hu) kits were obtained from USCN Life Science and Technology Company and IL-1β (Cat# 88-7013-88) kit from eBioscience to measure cytokine levels in both human plasma and lavage fluid. The 96-well-microplates were read using a VICTOR X3 plate reader.

### Statistical Analysis

Statistical analysis was performed using Prism 6.0 (GraphPad software) or SPSS 16.0. Data were expressed as mean ± SEM. Differences between experimental groups were tested using the unpaired *t*-test or one-way analysis of variance (ANOVA). *P* < 0.05 was considered statistically significant.

## Results

### Functional Derangement of BMDMs in T-Bet-Deficient Mice

**Figure 1:**
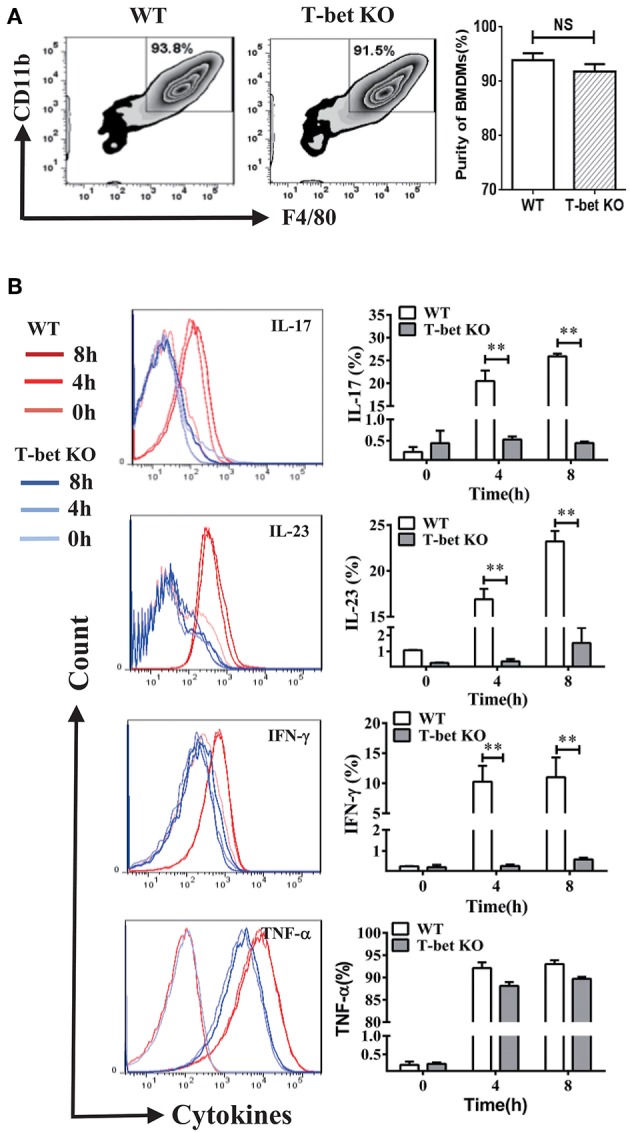
Functional derangement of BMDMs in T-bet KO mice. **(A)** BMDMs were identified by flow cytometry and the purity of those growth was comparable between T-bet KO and WT mice. **(B)** Levels of IL-17, IL-23, INF-γ, and TNF-α were analyzed using flow cytometry in BMDMs from T-bet KO mice and WT mice after MSU crystal challenge *in vitro*. Results are representative of 3 independent experiments. Each group had 3–5 mice **P < 0.01. BMDM, bone marrow-derived macrophage; IFN, interferon; IL, interleukin; KO, knockout; MSU, monosodium urate; TNF, tumor necrosis factor; WT, wild type.

The inflammatory cytokines released from macrophages by MSU-induced inflammation have capacity of recruitment and activation of inflammatory cells. To determine whether T-bet deficiency alters BMDM function, several cytokines were evaluated by flow cytometry at different time points after MSU crystal challenge. The BMDMs were identified by F4/80 and CD11b staining and their growth was comparable between T-bet KO and WT mice (Figure 1A). Compared with BMDMs from WT mice after MSU treatment *in vitro*, levels of inflammatory cytokines IL-17, IL-23 and INF-γ were reduced in T-bet KO mice, but the levels of TNF-α were not (Figure 1B). Thus, it is reasonable to speculate that the functional derangement of T-bet KO BMDMs may have an important role in the regulation of gout development.

### Reduction of MSU Crystal Phagocytosis in BMDMs With T-Bet Deficiency

**Figure 2:**
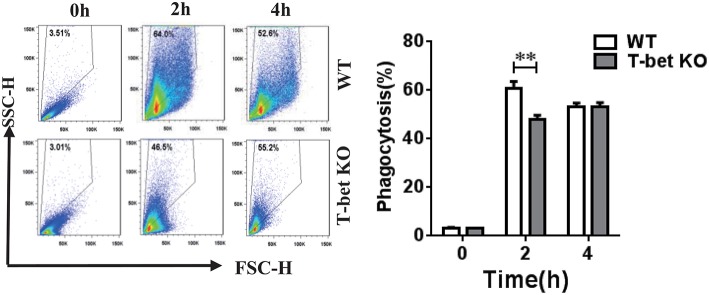
Reduction of MSU crystal phagocytosis in BMDMs from T-bet KO mice. MSU crystal phagocytosis was analyzed by flow cytometry in BMDMs from T-bet KO and WT mice at different time points after MSU crystal challenge *in vitro*. Results are representative of 3 independent experiments. *n* = 3–5 for each group and unpaired *t*-test was used for each group at indicated time points. **P < 0.01. BMDM, bone marrow-derived macrophage; KO, knockout; MSU, monosodium urate; WT, wild type.

MSU crystals are phagocytosed by macrophages and then trigger NLRP3 inflammasome activation, leading to IL-1β release (1, 2). A previous study from Xiao et al. (16) found that phagocytosis was enhanced in mouse macrophages that overexpress T-bet. To explore if the capacity of MSU crystal phagocytosis was modulated in the BMDMs with T-bet deletion, we performed a macrophage MSU crystal phagocytosis assay *in vitro*. WT and T-bet KO BMDMs were challenged with MSU crystals and analyzed for their SSC change, whose increase would represent phagocytosis of MSU crystals. MSU crystal phagocytosis in T-bet KO mice at 2 h after MSU crystal challenge was reduced compared with that in WT mice (Figure 2). It indicated that less release of cytokines from BMDMs in T-bet KO mice following MSU crystal challenge may be due to reduced MSU crystal phagocytosis.

### Alleviation of MSU-Induced Footpad Inflammation in T-Bet-Deficient Mice

**Figure 3:**
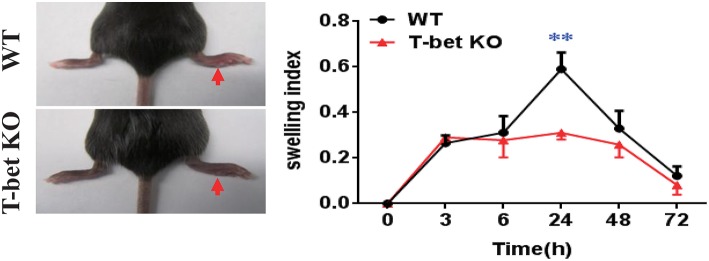
Alleviation of MSU-induced footpad inflammation in T-bet-deficient mice. One milligram/forty microliter MSU suspension was injected into the right footpad of WT and T-bet KO mice. Foot thickness was tested by an electronic caliper at the indicated time points (0, 3, 6, 24, 48, and 72 h) after MSU administration. Results are representative of 3 independent experiments. *n* = 3–5 for each group and unpaired *t*-test was used for each group at indicated time points. **P < 0.01. KO, knockout; MSU, monosodium urate; WT, wild type.

Based on the reduced inflammatory cytokines in T-bet KO mice, we examined whether the T-bet deficiency affected severity of MSU-induced gouty arthritis *in vivo*. An MSU-induced footpad model to mimic AG in humans was applied to evaluate the severity of inflammation. The swelling index was increased in WT mice after MSU crystal challenge and peaked at 24 h, whereas it was dramatically alleviated in T-bet KO mice, suggesting that deletion of T-bet expression could significantly affect the clinical phenotype of MSU-induced gouty inflammation *in vivo* (Figure 3).

### Weakened Inflammatory Cells Infiltration of MSU-Induced Peritonitis in T-Bet-Deficient Mice

**Figure 4:**
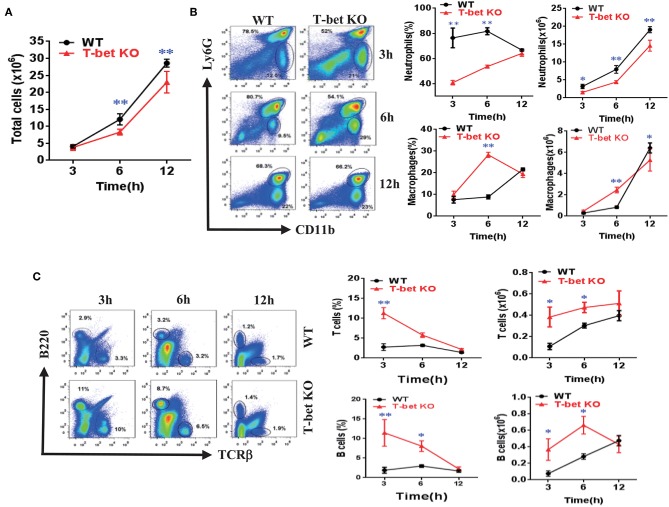
Weakened inflammatory cell infiltration of MSU-induced peritonitis in T-bet KO mice. **(A)** 3 mg/0.5 mL MSU suspension was injected into the peritoneal cavity, and the lavage fluid was harvested at different time points. Total cell number **(A)** was counted by a hematocytometer. **(B)** neutrophils and macrophages were analyzed by flow cytometry. Macrophages were represented by CD11b^+^, while neutrophils represented by Ly6G^+^. **(C)** T cells and B cells were analyzed by flow cytometry. T cells were represented by TCRβ^+^, and B cells by B220^+^. Results are representative of 3 independent experiments. *n* = 3–5 for each group and unpaired *t*-test was used for each group at indicated time points. *P < 0.05, **P < 0.01. KO, knockout; MSU, monosodium urate; WT, wild type.

To ascertain the function of T-bet deficiency in MSU-induced inflammation *in vivo*, a peritoneal cavity model was applied to assess the cellular phenotype of T-bet in gout. In comparison with WT mice, total cell number of peritoneal cavity lavage fluid (PCLF) was significantly decreased in T-bet KO mice at 6 and 12 h after MSU crystal challenge (Figure 4A). We next evaluated inflammatory profiles in the PCLF by flow cytometry. The infiltrated neutrophils were the majority of the peritoneal cell population (Figure 4B). In addition to comparable frequencies of neutrophils at 12 h, the absolute number and frequencies of neutrophils were dramatically decreased at other time points in T-bet KO mice (Figure 4B). Even though the number and frequency of macrophages increased dramatically at 6 h, the number of macrophages was decreased at 12 h in T-bet KO mice in spite of no significant changes in frequency. B and T cells from the peritoneal cavity exposed to MSU crystals were also analyzed by flow cytometry. The frequency of B and T cells, which became the minority population following MSU crystal challenge, was decreased in T-bet KO mice, whereas their number was increased and reached a comparable peak at 12 h (Figure 4C). These results suggest that T-bet ablation significantly weakens inflammatory cell infiltration, particularly neutrophils to inflammatory sites.

### Suppression of Inflammatory Cell Infiltration and Cytokine Release From MSU-Induced Air Pouch Model in T-Bet-Deficient Mice

**Figure 5:**
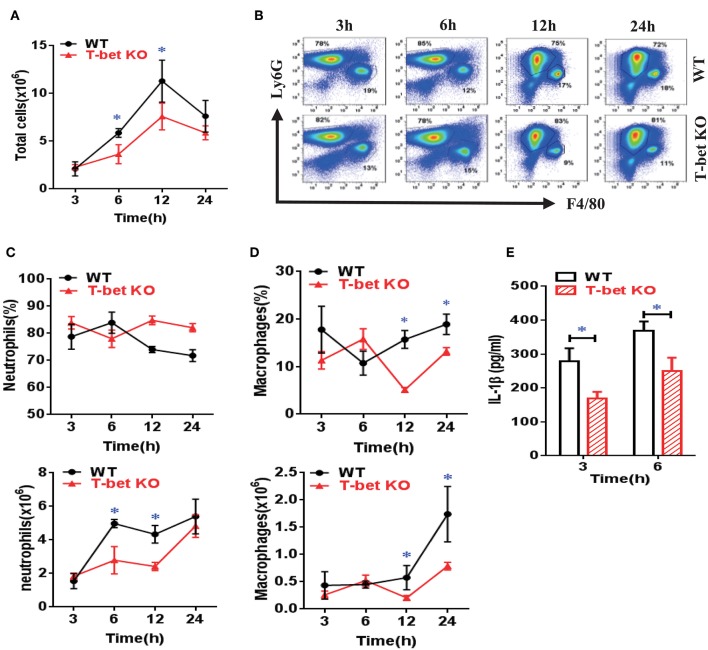
Suppression of inflammatory cell infiltration and cytokine release from MSU-induced air pouch model in T-bet-deficient mice. **(A)** total cell number was counted by a hematocytometer. **(B)** neutrophils and macrophages were gated by flow cytometry. Infiltrated macrophages were represented by F4/80^+^, and neutrophils by Ly6G^+^. **(C,D)** Infiltrated neutrophils **(C)** and macrophages **(D)** were analyzed using flow cytometry. **(E)** IL-1β protein levels in air pouch lavage fluid were measured at 3 and 6 h using enzyme-linked immunosorbent assays. Results are representative of 3 independent experiments. *n* = 3–5 for each group and unpaired *t*-test was used for each group at indicated time points. *P < 0.05. IL, interleukin; KO, knockout; MSU, monosodium urate; WT, wild type.

To clarify migration of inflammatory cells into inflammatory sites in T-bet-deficient mice, we examined inflammatory profiles in WT and T-bet KO mice using an air pouch model. As expected, the total cell number in the subcutaneous APLF from T-bet KO mice was markedly decreased compared with that from WT mice at 6 and 12 h after MSU injection (Figure 5A). The neutrophils and macrophages were migrated into the air pouch cavity upon MSU crystal stimulation (Figure 5B). Even though the frequency of neutrophils did not significantly change, the absolute number of neutrophils decreased dramatically at 6 and 12 h in T-bet KO mice (Figure 5C). Analogously, significant changes in frequency or number of macrophages were observed (Figure 5D). In comparison with WT mice, the cytokine IL-1β levels in APLF, particularly at the early phase of inflammation (3 and 6 h), were markedly reduced in T-bet KO mice after MSU crystal challenge (Figure 5E). Therefore, T-bet deficiency plays a vital role in relieving inflammatory cell infiltration and cytokine release.

### Downregulation of T-Bet Expression in Patients With AG

**Figure 6:**
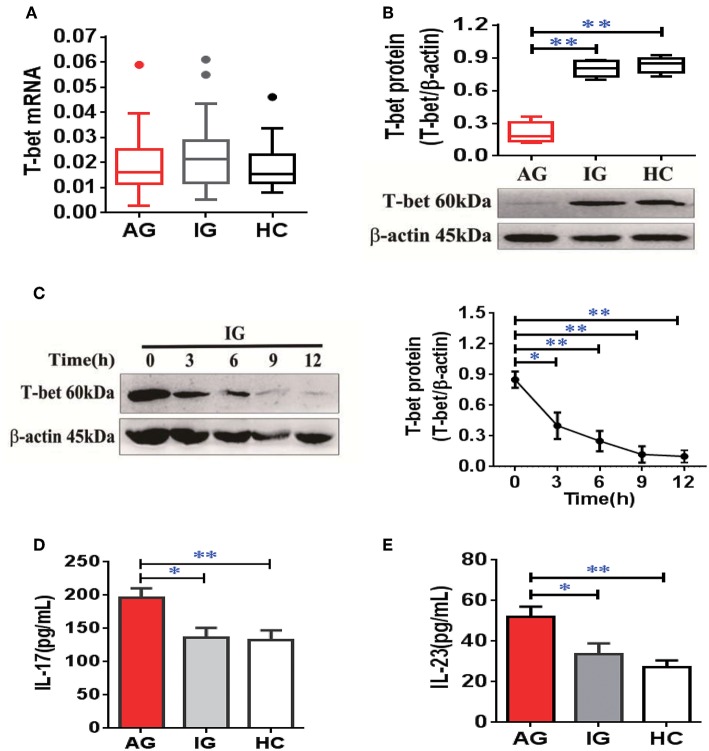
Downregulation of T-bet expression in PBMCs and elevation of inflammatory cytokines in plasma from AG patients. **(A,B)** mRNA **(A)** and protein **(B)** of T-bet were detected in PBMCs from patients with AG, IG, or HCs using quantitative real-time PCR or western blotting. **(C)** the protein levels of T-bet were further detected in PBMCs from IG patients (*n* = 4) exposed to MSU crystals at different time points. **(D,E)** Interleukin (IL)-17 **(D)** and IL-23 **(E)** levels were measured in patients with AG, IG, or HCs using enzyme-linked immunosorbent assays. AG (*n* = 45), IG (*n* = 42), or HCs (*n* = 42) for T-bet mRNA expression and inflammatory cytokines (IL-17 and IL-23) detection. *n* = 6 for T-bet protein expression in each group. One-way analysis of variance and the Bonferroni post-test were used for comparisons between groups. *P < 0.05, **P < 0.01. AG, acute gout; HC, healthy control; IG, intercritical gout; IL, interleukin; MSU, monosodium urate; PBMC, peripheral blood mononuclear cell.

To confirm expression of T-bet in patients with gout, the mRNA and protein levels of T-bet were detected. The mRNA levels of T-bet in PBMCs were comparable among AG, IG, and HCs (Figure 6A). However, the T-bet protein levels were significantly reduced in AG compared with IG or HCs (Figure 6B). In addition, the acute inflammatory response was triggered by MSU crystals in IG for different time points *in vitro*, and protein levels of T-bet after MSU crystal challenge were dramatically downregulated in a time-dependent manner (Figure 6C). We measured plasma levels of IL-17 and IL-23 in gout patients. The levels of IL-17 (Figure 6D) and IL-23 (Figure 6E) were significantly elevated in AG compared with IG patients or HCs. Collectively, downregulation of T-bet and elevation of inflammatory cytokines were observed in AG patients, demonstrating that T-bet could be actively involved in development of gouty inflammation.

## Discussion

To the best of our knowledge, this is the first study to explore that transcription factor T-bet is actively related to the development of gouty inflammation. Our findings demonstrate that ablation of T-bet protects against the phenotype of MSU-induced gouty inflammation in diverse murine models and T-bet is downregulated in patients with AG, suggesting that T-bet is required to regulate inflammation and plays a vital role in development of gouty inflammation.

The initial description of T-bet is a master regulator of commitment to the Th1 cell lineage. However, T-bet is now recognized as being related to the adaptive and innate immune systems (3). Development of gout is also related to the adaptive and innate immune systems. So far, there is a lack of direct evidence to verify that T-bet is involved in development of gouty inflammation. A recent review suggests that lacking T-bet protects against development of autoimmune diseases and production of inflammatory mediators (4). Therefore, T-bet deficiency could have capacity of controlling gout development.

We took advantage of T-bet KO mice to explore the T-bet function *in vitro* and *in vivo*. We found that T-bet KO reduced inflammatory cytokine production *in vitro*, but did not affect macrophage development, which could have been due to lack of T-bet expression in macrophages. Even so, overexpression of T-bet in mouse macrophages increases phagocytosis (16). Our results showed that T-bet deficiency decreased MSU crystal phagocytosis in BMDMs, suggesting that lack of T-bet affects macrophage phagocytosis. Thus, it is reasonable to explain that the functional derangement of T-bet KO BMDMs may play a role in the regulation of gout development.

The onset of CAIA in T-bet-deficient mice was delayed and was less severe in both the initial and the late phases (5). The footpad model of MSU-induced gouty inflammation in T-bet KO and WT mice was used to mimic AG in humans. Expectedly, the paw swelling index was significant alleviated in T-bet KO compared with WT mice, suggesting that T-bet deficiency does significantly improve the clinical phenotype of MSU-induced gouty inflammation.

A previous study (17) proved that the murine peritoneal cavity harbors a number of immune cells; the frequency of which is approximately 30% in macrophages, 50% in B cells and 10% in T cells. Upon stimulation with lipopolysaccharide, the neutrophils filtered into the peritoneum rapidly as well as macrophages/monocytes (18). Neutrophil influx into the synovium and joint fluid is one of the pathological hallmarks of AG (19–21). An acute inflammatory profile of peritonitis occurred in reaction to MSU crystals. Neutrophil and monocyte/macrophage migration to the peritoneum was observed after 4 h, and inflammatory cytokines, such as IL-1β, TNF-α, and IL-6, were elevated within 2 h and peaked at 4 h (22). Here, a murine peritoneal cavity model of MSU-induced inflammation was used to evaluate cellular phenotype of T-bet in gout. Mice with T-bet deficiency had fewer total cells in PCLF, which mostly comprised neutrophils and monocytes/macrophages. In contrast, the total number of T and B cells was increased in T-bet KO mice, although those became the minority population after MSU crystal challenge. The air pouch model, which mainly comprised recruitment of neutrophils and monocytes/macrophages, was created to rule out the complicated situations of peritoneal cavity cells. Analogously, T-bet deficiency dramatically relieved inflammatory cell infiltration and decreased inflammatory cytokine IL-1β levels. Therefore, all data are nearly consistent in the diverse models of MSU-induced gouty inflammation. It is in accordance with T-bet-deficient mice with different immune diseases such as CAIA, systemic lupus erythematosus, inflammatory bowel disease, and even AS (4, 8).

In addition to the above animal models, we measured T-bet expression in gout patients. We found that protein levels of T-bet were significantly downregulated in PBMCs from AG patients compared with IG patients or HCs, although the mRNA levels had no significant change among the 3 groups. Notably, a comparable level of T-bet protein was observed between IG patients and HCs. Furthermore, IG patients were treated with MSU crystals to mimic the acute gouty inflammatory response *in vitro*, Reduction of T-bet was observed in a time-dependent manner. These findings indicate that T-bet is downregulated in acute inflammation due to MSU crystal challenge and could be restored to the normal levels during remission of gouty inflammation. This is inconsistent with the increased T-bet expression in patients with AS (8), however, reduced levels of T-bet was observed in Kawasaki disease (23). Based on previous researches about T-bet's role in various diseases, most of the data are inconsistent, and incompletely understood. Here, Considering the elevated inflammatory cytokines in AG patients and reduced inflammation in T-bet KO mice following MSU crystal challenge, downregulation of T-bet in the acute inflammatory response could be a protective strategy for reducing inflammatory cell infiltration and cytokine release, and may contribute to spontaneous self-remission of gouty inflammation. More investigations should be performed to elucidate the mechanisms of T-bet involvement in regulation of gout development.

This study had some limitations. First, in addition to mice with T-bet deficiency, we did not include mice with T-bet overexpression to identify the effect of T-bet in the mouse model of gout. Second, the exact cellular mechanisms of T-bet related to regulation of gouty inflammation were not investigated, although there were some regulatory mechanisms of T-bet in autoimmune arthritis (24, 25). Third, a comparative study of diseases should be conducted because of the differential importance of T-bet in various diseases.

In conclusion, our results from humans and mice strongly suggest that transcription factor T-bet deficiency protects against MSU-induced gouty inflammation. Downregulation of T-bet could play a protective role to relieve the inflammation in AG.

## Data Availability

All datasets generated for this study are included in the manuscript and/or the Supplementary Files.

## Ethics Statement

All the subjects signed written informed consent forms. The study was conducted in accordance with the principles of the Declaration of Helsinki.

Handling of mice and experimental procedures were approved by Institutional Animal Care and Use Committee of Henry Ford Health System.

## Author Contributions

Q-BY and Y-LH performed most of the experiments. Q-BZ performed part of experiments. Q-BY, Q-SM, and J-GZ analyzed the data. Q-BY drafted the manuscript. Q-SM and J-GZ supervised the overall study and finalized the manuscript.

### Conflict of Interest Statement

The authors declare that the research was conducted in the absence of any commercial or financial relationships that could be construed as a potential conflict of interest.
